# Longitudinal survey on the distribution of *Biomphalaria sudanica* and *B. choanomophala* in Mwanza region, on the shores of Lake Victoria, Tanzania: implications for schistosomiasis transmission and control

**DOI:** 10.1186/s13071-017-2252-z

**Published:** 2017-06-28

**Authors:** Anouk N. Gouvras, Fiona Allan, Safari Kinung’hi, Muriel Rabone, Aidan Emery, Teckla Angelo, Tom Pennance, Bonnie Webster, Honest Nagai, David Rollinson

**Affiliations:** 1 Department of Life Sciences, Natural History Museum, Cromwell Road, London, SW7 5BD UK; 2 National Institute for Medical Research (NIMR) Mwanza Centre, P.O Box 1462, Mwanza, United Republic of Tanzania; 3 London Centre for Neglected Tropical Disease Research, London, UK

**Keywords:** *Biomphalaria*, *B. sudanica*, *B. choanomphala*, Mwanza, Lake Victoria, *Schistosoma mansoni*, Intestinal schistosomiasis, Transmission, Seasonality, Snail

## Abstract

**Background:**

Schistosomiasis is hyper-endemic in the Lake Victoria basin; with intestinal schistosomiasis plaguing communities adjacent to the lake, where the intermediate host snails live. The two intermediate host species of *Schistosoma mansoni* in the Mwanza region are *Biomphalaria sudanica*, found on the banks of the lakes, and *B. choanomphala*, found in the lake itself. There are few longitudinal surveys documenting changing abundance and differential transmission patterns of these *Biomphalaria* snails across seasons and years. We undertook 15 field surveys at 26 sites over four years to determine the parameters that influence *Biomphalaria* abundance, presence of *S. mansoni*-shedding snails and impact of schistosomiasis treatment interventions on transmission potential in the Mwanza region.

**Results:**

Statistical analysis revealed seasonal difference in the abundance of *B. sudanica* with the highest number of snails found in the dry season (Kruskal-Wallis *χ* ^2^ = 37.231, *df* = 3, *P* < 0.0001). Water measurements were not associated with *B. sudanica* abundance; however, high levels of rainfall did have a negative effect on *B. sudanica* [coefficient effect -0.1405, 95% CI (-0.2666, -0.0144)] and *B. choanomphala* abundance [coefficient effect -0.4388, 95% CI (-0.8546, -0.0231)] potentially due to inundation of sites “diluting” the snails and influencing collection outcome. *Biomphalaria sudanica* snails were found at all sites whereas *B. choanomphala* were far more focal and only found in certain sites. Shedding *Biomphalaria* did not show any variation between dry and rainy seasons; however, a decrease in shedding snails was observed in year 4 of the study.

**Conclusions:**

*Biomphalaria sudanica* is uniformly present in the Mwanza region whereas *B. choanomphala* is far more focal. Seasonality plays a role for *B. sudanica* abundance, likely due to its habitat preference on the banks of the lake, but not for *B. choanomphala*. The decrease in shedding *Biomphalaria* abundance in Year 4 could be linked to ongoing schistosomiasis treatment efforts in the neighbouring human populations. The highest number of shedding *Biomphalaria* was observed at sites with high levels of human movement. Prioritising snail control at such sites could greatly reduce transmission in these high-risk areas.

**Electronic supplementary material:**

The online version of this article (doi:10.1186/s13071-017-2252-z) contains supplementary material, which is available to authorized users.

## Background

Schistosomiasis is a Neglected Tropical Disease (NTD) affecting over 200 million people worldwide with an at-risk population of 700 million people [1]. The aetiology of this disease is a small blood-dwelling digenetic trematodes of the genus *Schistosoma*, characterised by a complex, obligate indirect life-cycle, involving an intermediate aquatic snail host and transmission through direct water contact. The geographical range of schistosomes is restricted by climatic factors influencing the ecological habitat of their intermediate host snails [2].

*Schistosoma mansoni* infects over 83 million people [1, 3] across sub-Saharan Africa, the Middle East, parts of South America and the Caribbean Islands [4]. It causes intestinal schistosomiasis potentially resulting in severe liver fibrosis, spleen damage and pulmonary hypertension [5]. *Schistosoma mansoni* parasites utilize species of *Biomphalaria* as the intermediate snail host [6]. Several species of both genera are involved in *Schistosoma* infections across sub-Saharan Africa [6].

According to the 2012 population census the population of Tanzania was 43 million [7], although it is estimated that the current population is closer to 55 million. Currently the entire population of Tanzania including the Zanzibar Archipelago are at risk of schistosome infections [8] with an estimated prevalence of 53.3% [9]. Arguably the most at-risk human populations live in the Lake Victoria Basin, an area spanning Tanzania, Uganda, Kenya, Rwanda and Burundi supporting over 35 million people [10]. Lake Victoria whose shores are bordered by Tanzania, Uganda and Kenya, is hyper-endemic for schistosomiasis with all three countries reporting mid to high prevalence, particularly for fishing communities in the area [11–13].

In the Mwanza region, both urogenital and intestinal schistosomiasis occur: urogenital schistosomiasis occurs primarily in remote areas away from the Lake Victoria shore while intestinal schistosomiasis occurs along the shore [14]. The two species of *Biomphalaria* found in the Mwanza region of Lake Victoria are *Biomphalaria sudanica* and *B. choanomphala*. The former is found in the shallow vegetation on the banks of the lakes or in the marshes adjacent to the lake whereas *B. choanomphala* is found in the lake itself [15, 16] although whether these are separate species or merely ecophenotypes is still disputed [15–17] (for this study we refer to *B. sudanica* and *B. choanomphala* as separate species though we do recognise that this is not set in stone). Both species are involved in *S. mansoni* transmission although their involvement may vary from site to site. Whilst there have been several excellent studies on factors influencing the distribution of these snail species [15, 17–19] as well as development of GIS and Bayesian models for schistosomiasis surveillance based on suitable snail habitat and schistosome transmission [20, 21], there are numerous gaps of knowledge on the changing distribution of *Biomphalaria* species of Lake Victoria. In particular, there is a lack of longitudinal survey data across years and seasons.

In this study, we report on longitudinal surveys of *Biomphalaria* species in the Mwanza region of Lake Victoria, Tanzania. The study was implemented in the context of the larger scale gaining and sustaining control of schistosomiasis studies of the Schistosomiasis Consortium for Operational Research and Evaluation (SCORE) project [22]. The aim of this study was to investigate and quantify the factors related to snail-human infection processes within the context of School Based Treatment (SBT) and Community Based Treatment (CBT) mass drug administration (MDA) strategies [22]. Specifically, the study conducted surveys to identify *S. mansoni* transmission sites and to determine the intermediate snail hosts (*Biomphalaria* spp.) at these sites and the parameters that influence intermediate snail host abundance and disease transmission potential.

## Methods

### Study area

#### Climatic factors

Temperature and precipitation data were acquired from the United States Department of Agriculture Foreign Agricultural Service (data collected from Tanzania Meteorological Agency http://gis.pecad.fas.usda.gov/WmoStationExplorer/). Using daily temperature and precipitation data from the Mwanza station, mean monthly minimum, maximum and average temperatures and total monthly precipitation were calculated. To determine climate temperature and precipitation for each collection record, the monthly average temperature for the sampling date and the sum of the total amount of precipitation 28 days prior to the sampling date per site were determined. The latter was chosen because snail eggs take at least 4 weeks to reach maturity [2].

#### *Biomphalaria* snail surveys

A total of 15 snail collection surveys were undertaken in the Mwanza region along the banks of Lake Victoria and at two villages in the neighbouring Geita region between January 2012 and December 2015. The Mwanza region spans an area of 9467 km^2^ with an estimated population of 2,772,509 people [7]. *Schistosoma mansoni* is the main schistosome species in the Mwanza region, with transmission occurring in the lake or in marshy habitats adjacent to the lake. *Schistosoma haematobium*, although less prevalent, is also present in the region; however, source of infection for this species seems to occur in smaller water bodies away from the lake [23]. Lake Victoria is characterised by stable constant temperatures and two rainy seasons; the Masika (long rains) from March through to May and Vuli (short rains) occurring in October through to January. A dry season of no or little rain occurs from June through to September each year with January and February acting as a transitional season.

**Fig. 1:**
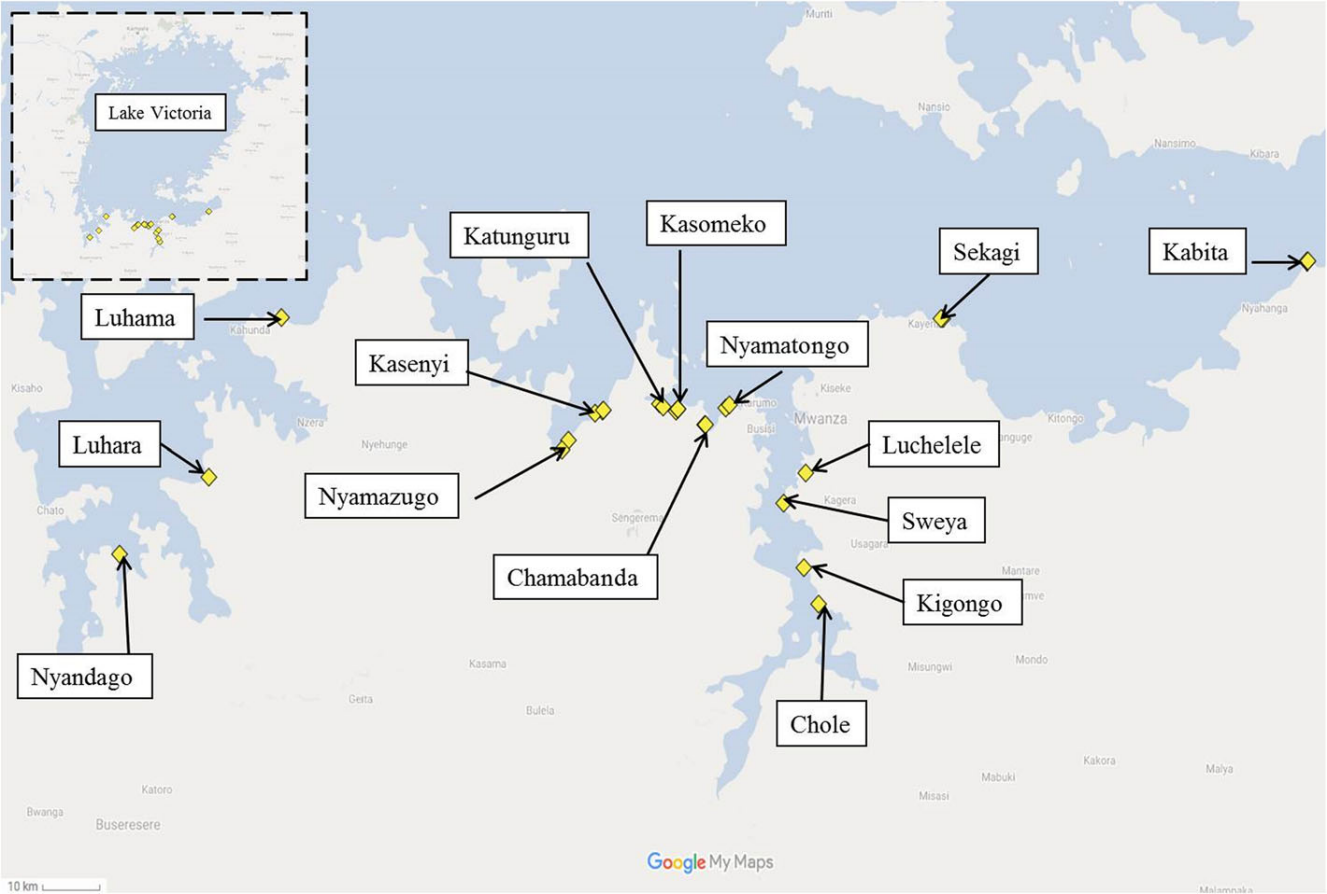
Map showing *Biomphalaria* survey sites in Tanzania

Snail survey sites were chosen based on 15 randomly selected schools participating in the main SCORE project and were identified using local information on water based activities (bathing, fishing, water collection). For this longitudinal study, 26 snail survey sites in these 15 villages were surveyed. Figure 1 shows the location of these snail sites and Table 1 gives additional information on the habitat type and the nearest village name. As stated before, these villages were part of a larger scale SCORE project for gaining and sustaining control of schistosomiasis and as such were undergoing different mass drug administration (MDA) strategies [22]. The MDA strategy of the nearest village is also recorded in Table 1.

**Table 1:** SCORE snail sites included in the longitudinal surveys (*n* = 15)

District	Village name	Site	Site type	Mass Drug Administration intervention strategy
Geita	Luhara	Luhara	Lake	Biennial school-based
Geita	Nyandago	Nyandago	Marsh on lake shore	Annual community-wide
Magu	Kabita	Kabita 1	Marsh behind lake shore	Biennial school-based
Magu	Kabita	Kabita 2	Marsh behind lake shore	Biennial school-based
Magu	Kabita	Kabita 3	Marsh behind lake shore	Biennial school-based
Magu	Sekagi	Sekagi 1	Marsh behind lake shore	Annual community-wide
Magu	Sekagi	Sekagi 2	Marsh behind lake shore	Annual community-wide
Misungwi	Isamilo	Chole	Marsh on lake shore	Biennial school-based
Misungwi	Bukumbi	Kigongo	Lake	Annual community-wide
Nyamagana	Luchelele	Luchelele	Lake	Biennial school-based
Nyamagana	Sweya	Sweya	Marsh and rice paddy	Annual school-based
Sengerema	Chamabanda	Chamabanda 1	Marsh on lake shore	Biennial school-based
Sengerema	Chamabanda	Chamabanda 2	Marsh on lake shore	Biennial school-based
Sengerema	Kasenyi	Kasenyi	Marsh on lake shore	Two years’ community-wide then two years’ school-based
Sengerema	Kasenyi	Kasenyi/Mikoloshini	Marsh on lake shore	Two years community-wide then two years school-based
Sengerema	Kasenyi	Kasenyi/Nyamahona	Marsh on lake shore	Two years community-wide then two years school-based
Sengerema	Kasomeko	Kasomeko 1	Lake	Annual community-wide
Sengerema	Kasomeko	Kasomeko 2	Lake and rice paddy	Annual community-wide
Sengerema	Kasomeko	Kasomeko 3	Lake	Annual community-wide
Sengerema	Katunguru	Katunguru 1	Marsh on lake shore	Biennial school-based
Sengerema	Katunguru	Katunguru 2	Lake	Biennial school-based
Sengerema	Luhama	Luhama	Marsh on lake shore	Two years’ community-wide
Sengerema	Nyamatongo	Nyamatongo 1	Marsh on lake shore	Annual community-wide
Sengerema	Nyamatongo	Nyamatongo 2	Marsh on lake shore	Annual community-wide
Sengerema	Nyamazugo	Nyamazugo 1	Lake	Biennial school-based
Sengerema	Nyamazugo	Nyamazugo 2	Mash on lake shore	Biennial school-based

*Abbreviation: MDA* Mass Drug Administration Programme

Each site was no further than 5–10 m from the lakeshore, no more than 15 m in length and was identified by the village/school name and ‘1’, ‘2’, ‘3’ where more than one site per school was used. The geographical coordinates of the schools and their respective snail sites were recorded on a hand held Geographical Positioning System (Garmin eTrex, Taiwan).

*Biomphalaria* snails were collected semi-quantitatively by scooping using handheld metal sieve scoops (25 cm diameter, 0.5 mm mesh) for 15 min and hand collection with forceps (2 people scooping and 2 people collecting the scooped content into a collection jar) on the banks of Lake Victoria, and by dredging using a metal dredge (diameter 50 cm, 1 mm mesh) dropped by boat approximately 10 m into the lake and then dragged back to shore, to look for the deeper living *Biomphalaria* species.

At each lakeshore site water level was noted (flooded, normal or low), a water sample was collected and water temperature, conductivity, total dissolved solids, salinity and pH were recorded as potential variables that influence snail abundance in freshwater habitats [6]. All water chemistry variables were recorded on a Thermo Scientific Eutech Multiparameter PCTEST35K handheld meter (Fisher Scientific UK Ltd.®, Loughborough, UK).

In addition, presence or absence of human activity such as bathing, washing clothes, fishing and agricultural practices were recorded as well as fauna and flora (including other snail species - molluscs) and vegetation (both wild and agricultural) were recorded. All collected *Biomphalaria* were taken back to the laboratory and putatively identified to species level using morphological traits of the shell [24]. The abundance (number) of each *Biomphalaria* species at each site was recorded. All *Biomphalaria* snails were placed in 24-well ELISA plates and put under light to induce cercarial shedding on the day of collection and again the following day [2]. Cercariae were morphologically identified as *S. mansoni* and stored on Whatman® FTA Cards, a subset were sequenced (*cox*1 and ITS) to confirm cercariae identification [16]. All infected snails and up to 50 non-shedding *Biomphalaria* snails from each site were fixed in ethanol (100%). These snail and cercariae samples were entered into the Schistosomiasis Collection at the Natural History Museum, London [25].

### Statistical analysis

Statistical analyses were undertaken using the R statistical software version 3.3.2 [26] and Rstudio version 0.99.491 [27].

#### *Biomphalaria* abundance analysis

##### Comparison of *Biomphalaria* abundance between seasons

Does *Biomphalaria* abundance show seasonal variation? More specifically is there a significant difference between the average abundance of *Biomphalaria* snails across all sites by season?

To address this question the snail abundance data were first split into the following groups: (i) collected by dredge and scoop (*Biomphalaria*); (ii) collected by scoop (*Biomphalaria sudanica*); (iii) collected by dredge (*Biomphalaria choanomphala*). Because snail abundance data are not normally distributed, the variance is greater than the mean and variance may be different in each group, two non-parametric tests, Kruskal-Wallis test and Welch’s ANOVA, were used to compare medians among group.

##### *Biomphalaria* species abundance analysis using monthly temperature, accumulative precipitation and water properties

We used Generalised Linear Mixed Model (GLMM); an extension of linear regression models which can accommodate error distribution from a variety of probability distributions (Poisson/Gaussian, binomial, negative binomial, gamma) and can account for random effects violating assumption of independence of samples often due to sampling design, e.g. repeated collections from same site or year or survey. GLMM can also handle unbalanced sample sizes. First, the data were explored to determine outliers (using Cleveland dotplots and boxplots), collinearity (pairplots and GVIF values) and relationships between covariates (multi-panel scatterplots) [28]. We used a GVIF value of over 3 as indicative of collinearity between variables [28]. Explanatory variables were standardised (centred on the mean and divided by the standard deviation) and scaled to enable comparison of fixed effect sizes. To fit the GLMMs we used the R package ‘*glmmadmb*’ [29, 30] with site and survey number as random effects. Because water property data could only be collected for scooping sites we only used these measurements for *B. sudanica* abundance analysis. Weather properties (temperature and precipitation), water levels and other observations at each site (presence and absence of human activity, of other molluscs and animals) and GPS coordinates were used for both *B. sudanica* and *B. choanomphala* analyses. Models were compared and chosen based on their Akaike’s Information Criterion (AIC) and negative Log-likelihood values.

Initial analysis for *B. sudanica* data indicated over-dispersion due to the large variation in the *Biomphalaria* count data, therefore a negative binomial GLMM was fitted. Data exploration for *B. choanomphala* data revealed high number of zeros which is to be expected with these deeper dwelling, harder to sample snails, so zero-inflation and hurdle models were used. These two model types treat high number of zeros differently. Zero-inflation models assume that the zeros from the data may be due to several reasons such as study design flaws, observation errors or unsuitable conditions or survey sites. In this study we used a standard GLMM with a negative binomial distribution and a zero-inflation parameter as described described in the ‘*glmmadmb*’ package [29, 30]. Conversely hurdle models first assume a binary outcome based on the Bernoulli probability on whether the outcome is zero (FALSE) or greater than zero (TRUE), using a binomial GLMM. The ‘TRUE’ count data is then analysed using a zero-truncated quasi-Poisson GLMM. The hurdle model structure therefore assumes that there are conditions that need to be met for *B. choanomphala* snails to be present and then once these conditions are met, (passing the hurdle) analysis can be done on the *B. choanomphala* zero-truncated count data.

#### Schistosoma mansoni-shedding *Biomphalaria* analysis

##### Comparing prevalence of shedding *Biomphalaria* by season, village and treatment strategy

Does the abundance of shedding *Biomphalaria* snails differ between sites, seasons and years? And do treatment interventions taking place in the local community influence the number of shedding *Biomphalaria*? These were tested using two non-parametric tests, Kruskal-Wallis test and Welch’s ANOVA.

##### Shedding *Biomphalaria* species analysis using weather properties, human activity, habitat and year

Explanatory variables used for shedding *Biomphalaria* were weather properties (temperature and precipitation), abundance of *B. sudanica* and abundance of *B. choanomphala*, GPS coordinates, human activity at the snail collection sites and year of collection. Shedding *Biomphalaria* species were analysed collectively to ensure good sample size. Shedding *Biomphalaria* abundance (count data) and presence/absence of shedding *Biomphalaria* data were analysed using a zero-inflated negative binomial GLMM and a binary binomial GLMM, respectively. Models were compared and chosen based on their AIC and negative Log-likelihood values.

## Results

### Results of snail surveys

In total 42,874 *Biomphalaria* snails were collected across the 26 sites over 4 years. Of these 42,816 were identified either as *B. sudanica* or as *B. choanomphala* with 58 *Biomphalaria* not identified to the species level. Those not identified to species level were from one site survey (Luhara, March 2012) which was due to a recording error. These entries were included in the analysis of all *Biomphalaria* but excluded in analysis done for specific species. In total 509 (1.18%) *Biomphalaria* were found to be shedding *S. mansoni* cercariae. Of these, 439 (1.22%) *B. sudanica* and 61 (0.88%) *B. choanomphala* were shedding *S. mansoni* cercariae (Table 2).

**Table 2:** Total number of *Biomphalaria* snail species collected and number of those shedding cercariae by survey

Survey (Month-Year)	*Biomphalaria*		*B. sudanica*		*B. choanomphala*	
	Collected	Shedding (%)	Collected	Shedding (%)	Collected	Shedding (%)
Jan-12	1060	36 (3.37)	1026	36 (3.51)	34	0 (0)
Mar-12	2136	26 (1.22)	1578	22 (1.39)	500	4 (0.80)
Jul-12	4889	111 (2.27)	3505	98 (2.80)	1384	4 (0.29)
Nov-12	2905	40 (1.38)	2363	36 (1.52)	542	4 (0.74)
Mar-13	2571	57 (2.22)	2336	49 (2.10)	235	8 (3.40)
Jul-13	3721	21 (0.56)	2248	11 (0.50)	1473	10 (0.68)
Oct-13	3166	38 (1.20)	3128	38 (1.21)	38	0 (0)
Feb-14	2537	29 (1.14)	2397	20 (0.83)	140	9 (6.43)
May-14	2756	46 (1.67)	1784	36 (2.02)	972	10 (1.03)
Sep-14	3536	14 (0.40)	3232	14 (0.43)	304	0 (0)
Dec-14	2285	19 (0.83)	2102	17 (0.81)	183	2 (1.09)
Feb-15	2893	20 (0.70)	2349	18 (0.77)	544	2 (0.37)
May-15	1613	36 (2.23)	1325	29 (2.19)	288	7 (2.43)
Aug-15	3673	8 (0.22)	3505	7 (0.20)	168	1 (0.59)
Nov-15	3133	8 (0.25)	3032	8 (0.26)	101	0 (0)
Total	42,874	509 (1.19)	35,910	439 (1.21)	6906	61 (0.88)

Numbers will not match total *Biomphalaria* due to 58 snails with missing species data

### Climate and seasonality

**Fig. 2:**
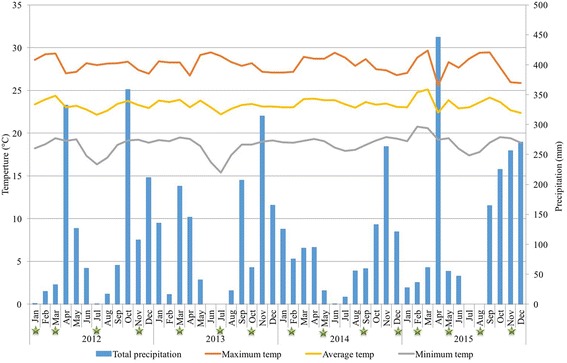
Total monthly precipitation, average monthly temperature and month of snail survey in Mwanza. Based on data from United States Department of Agriculture Foreign Agricultural Service (collected from Tanzania Meteorological Agency, Mwanza Station). Source: http://gis.pecad.fas.usda.gov/WmoStationExplorer/. Green stars indicate when a snail survey was undertaken

Figure 2 shows the typical bimodal tropical climate of Mwanza characterised by stable annual temperatures and two rainy seasons (Masika and Vuli rains) and one dry season. Also indicated on Fig. 2 is the month of each snail collection survey across the 4 years.

### *Biomphalaria* abundance analysis

#### Comparison of average snail abundance between seasons

**Fig. 3:**
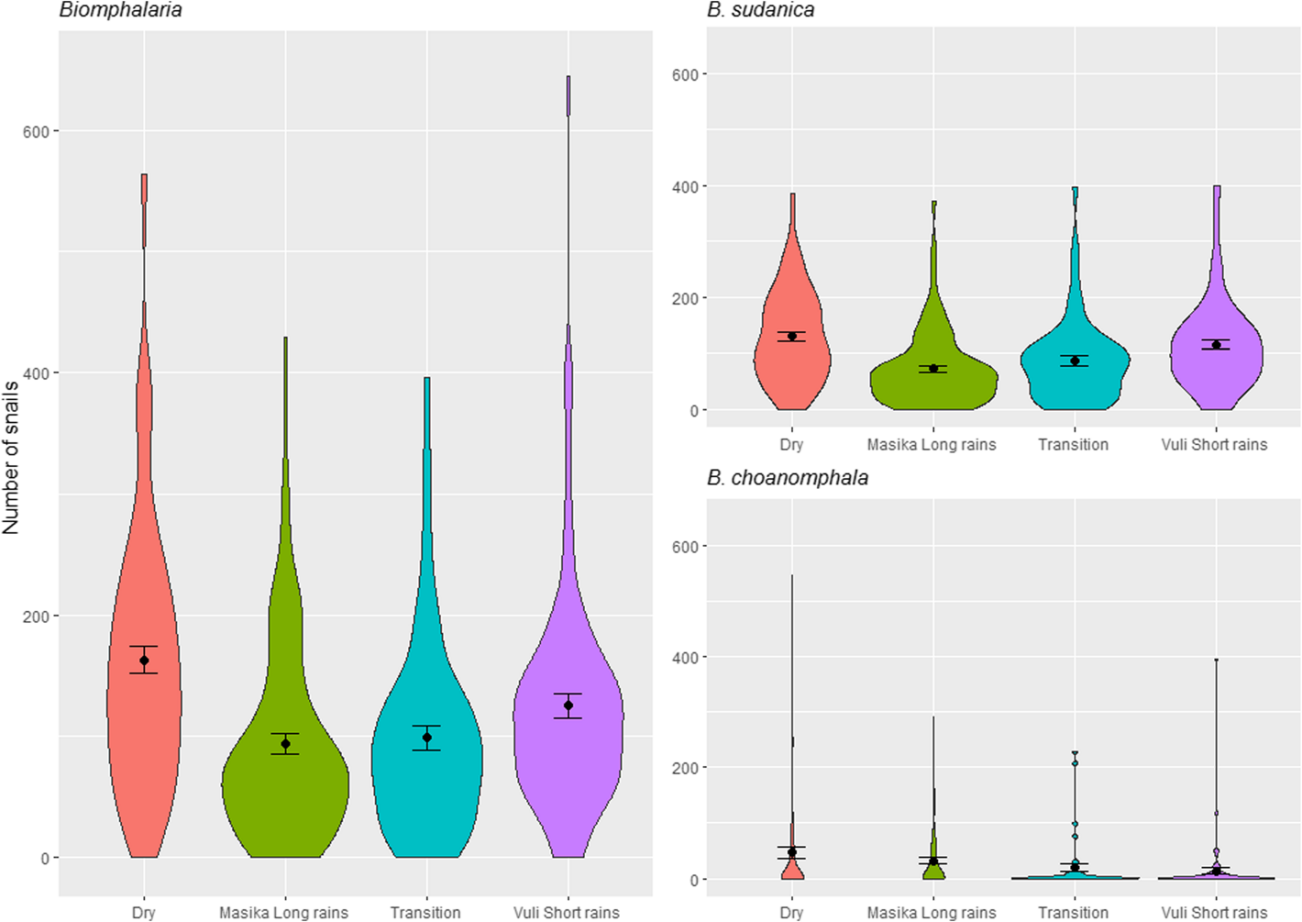
Violin plots including mean and standard error of mean of *Biomphalaria* snails (all), *B. sudanica* and *B. choanomphala* collected by season

Figure 3 suggests potential seasonality in snail abundance with highest number of snails collected in the dry season (June–September). Seasonal median abundance of *Biomphalaria* was found to be significantly different (Kruskal-Wallis *χ* ^2^ = 33.893, *df* = 3, *P* < 0.0001). A *post-hoc* test indicated that the dry season median differed significantly from the Masika long rains median and the transitional season median as did Masika Long rains from Vuli short rains. However, differences between dry and Vuli short Rains, Masika long rains and transitional and transitional & Vuli short rains were not significant (Kruskal-Wallis *post-hoc* testing, *P* > 0.05). Because our data violated the assumption of homogeneity of variance we also tested for seasonal difference in mean *Biomphalaria* abundance using a one-way ANOVA with a Welch correction for non-homogeneity. Welch’s ANOVA confirmed the findings of the Kruskal-Wallis test (*F*_(3, 186.98)_ = 9.4044, *P* < 0.0001).

Looking at the impact of season on *B. sudanica* and *B. choanomphala* abundance (Fig. 3) there is a significant impact of season on the abundance of *B. sudanica* (Kruskal-Wallis *χ* ^2^ = 38.734, *df* = 3, *P* < 0.0001) but not for *B. choanomphala* (Kruskal-Wallis *χ* ^2^ = 6.542, *df* = 3, *P* = 0.08803). *Post*-*hoc* multiple comparison tests indicated that *B. sudanica* median abundance was significantly different between the Dry season and Masika long rains, Dry season and Transition and between the Masika long rains and the Vuli short rains. But no difference was found between Masika and Transition, Dry and Vuli or Transition and Mvuli (Kruskal-Wallis *post-hoc* testing, *P* > 0.05). Welch’s ANOVA confirmed the findings of the Kruskal-Wallis test for *B. sudanica* (*F*_(3, 183.55)_ = 11.752, *P* < 0.0001) and *B. choanomphala* (*F*_(3, 115.12)_ = 2.417, *P* = 0.070).

#### Monthly and yearly variations in snail abundance

**Fig. 4:**
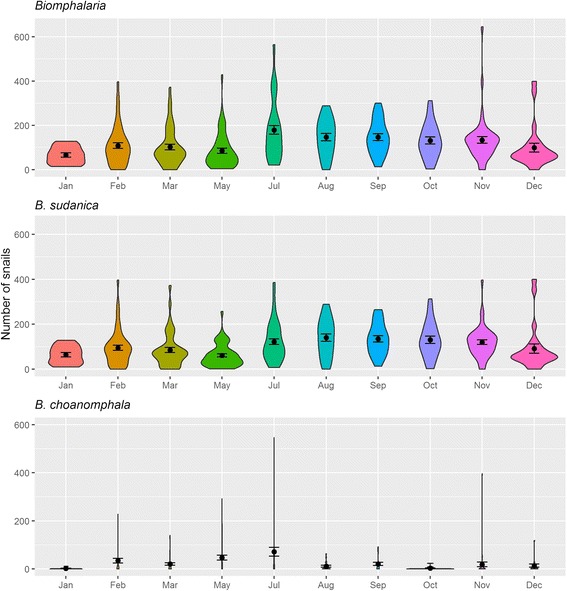
Violin plots including mean and standard error of mean of *Biomphalaria* snails (all), *B. sudanica* and *B. choanomphala* collected by month (aggregated across years)

*Biomphalaria sudanica* and *B. choanomphala* abundance (Fig. 4) varies by month (Kruskal-Wallis *χ* ^2^ = 52.623, *df* = 9, *P* < 0.0001 and Kruskal-Wallis *χ* ^2^ = 22.005, *df* = 9, *P* = 0.009, respectively). Welch’s ANOVA confirmed the findings of the Kruskal-Wallis test for *B. sudanica* and *B. choanomphala* (*F*_(9, 114.5)_ = 6.786, *P* < 0.0001 and *F*_(9, 75.177)_ = 4.578, *P* < 0.0001). Whereas no significant difference was found in yearly abundance of *Biomphalaria* snails (Kruskal-Wallis *χ* ^2^ = 1.431, *df* = 3, *P* = 0.698).

#### *Biomphalaria sudanica*

Strong collinearity was found between TDS, conductivity and salinity, some weak collinearity was found between average monthly precipitation and accumulative precipitation 4 weeks before samples. GLMMs were run with only one of the dissolved ion water measurements (water TDS) and one measurement of precipitation. The GLMM with the best fit based on AIC and negative Log-likelihood included average monthly temperature and observed water levels at site (Additional file 1: Table S1) although a GLMM with accumulative precipitation 4 weeks before sampling rather than observed water level also fit the data well (Additional file 1: Table S1).

The GLMMs indicate that increased precipitation has a negative association with abundance of *B. sudanica*, whether this is comparing flooded water level at sites to normal water level at sites [normal water level coefficient effect 0.405, 95% CI (0.107, 0.704)] or 4 weeks accumulative precipitation [coefficient effect -0.143, 95% CI (-0.271, -0.016)]. GLMM 2 (Additional file 1: Table S1) also shows a negative association with increasing average temperature [coefficient effect -0.193, 95% CI (-0.324, -0.062)] and with an interaction between average temperature and 4 weeks accumulative precipitation [coefficient effect -0.166, 95% CI (-0.314, -0.017)].

#### *Biomphalaria choanomphala*

The zero-inflated negative binomial GLMM with the best fit based on AIC and negative Log-likelihood included 4-weeks accumulative precipitation and districts as explanatory variables (Additional file 1: Table S2). The best hurdle GLMMs (binary binomial GLMM and zero-truncated GLMM) had 4-weeks accumulative precipitation (Precip4WT) and longitude and latitude coordinates as explanatory variables (Additional file 1: Table S3).

The zero-inflated GLMM (Additional file 1: Table S2) indicates that increased precipitation has a negative association with abundance of *B. choanomphala* [Precip4WT coefficient effect: -0.433, 95% CI (-0.859, -0.016)]. Magu and Misungwi showed a strong negative association with *B. choanomphala* abundance [Magu coefficient effect: -5.330, 95% CI (-7.043, -3.612); Misungwi coefficient effect: -3.952, 95% CI (-5.776, -2.128)] compared to Geita district.

The zero-truncated GLMM (Additional file 1: Table S3) indicates that increased precipitation has a negative association with abundance of *B. choanomphala* [Precip4WT coefficient effect: -0.281, 95% CI (-0.492, -0.070)]. Latitudinal measurements showed a strong positive association with *B. choanomphala* abundance [coefficient effect: 1.888, 95% CI (0.314, 3.463)] whereas longitudinal measurements had a negative association [coefficient effect: -0.992, 95% CI (-1.422, -0.562)]. In the binomial GLMM (Additional file 1: Table S3), longitudinal measurements were negatively associated with the presence of *B. choanomphala* [coefficient effect: -2.533, 95% CI (-3.979, -1.086)].

### *Schistosoma mansoni*-shedding *Biomphalaria* analysis

#### Shedding *Biomphalaria* by season and village

**Fig. 5:**
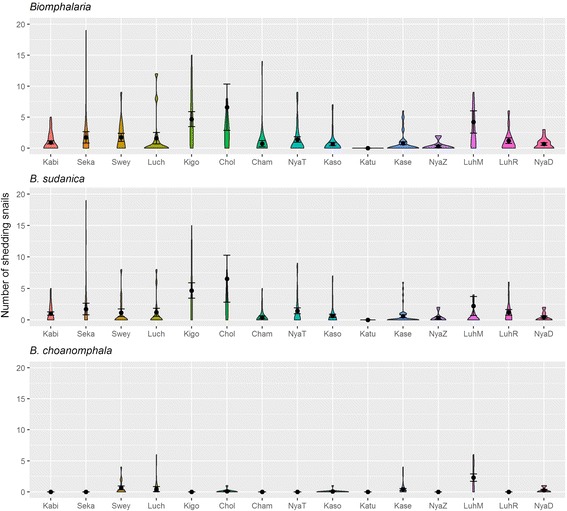
Violin plots including mean and standard error of mean of *S. mansoni*-shedding *Biomphalaria* snails (all)*, B. sudanica* and *B. choanomphala* snail species collected by village (aggregated across years). *Abbreviations*: Kabi, Kabita; Seka, Sekagi; Swey, Sweya; Luch, Luchelele; Kigo, Kigongo; Chol, Chole; Cham, Chamabanda; NyaT, Nyamatongo; Kaso, kasomeko; Katu, Katunguru; Kase, Kasenyi; NyaZ, Nyamazugo; LuhM, Luhama; LuhR, Luhara; NyaD, Nyandago. *Outliers not shown: 58 shedding snails from Chole and 27 shedding snails from Luhama

There was no significant difference in the number of *Biomphalaria* snails shedding *S. mansoni* cercariae between seasons (Kruskal-Wallis *χ* ^2^ = 1.357, *df* = 3, *P* = 0.716) or month (Kruskal- Wallis *χ* ^2^ = 12.963, *df* = 9, *P* = 0.1643). In Fig. 5 the number of *Biomphalaria* snails shedding cercariae at each village is shown. Shedding snails were found at most villages apart from the Katunguru sites.

The mean abundance of shedding *B. sudanica* snails was highest at Kigongo and Chole, both characterised by high levels of human water contact activities (ferry and fishing). *Biomphalaria choanomphala* shedding snails were found at 6 sites: Luhama, Sweya, Luchelele, Kasenyi, Nyandago, Chole and Kasomeko. Luhama, a busy fishing beach, had the highest abundance of shedding *B. choanomphala*.

#### Shedding *Biomphalaria* in annual community-wide treatment and biennial school-based treatment

**Fig. 6:**
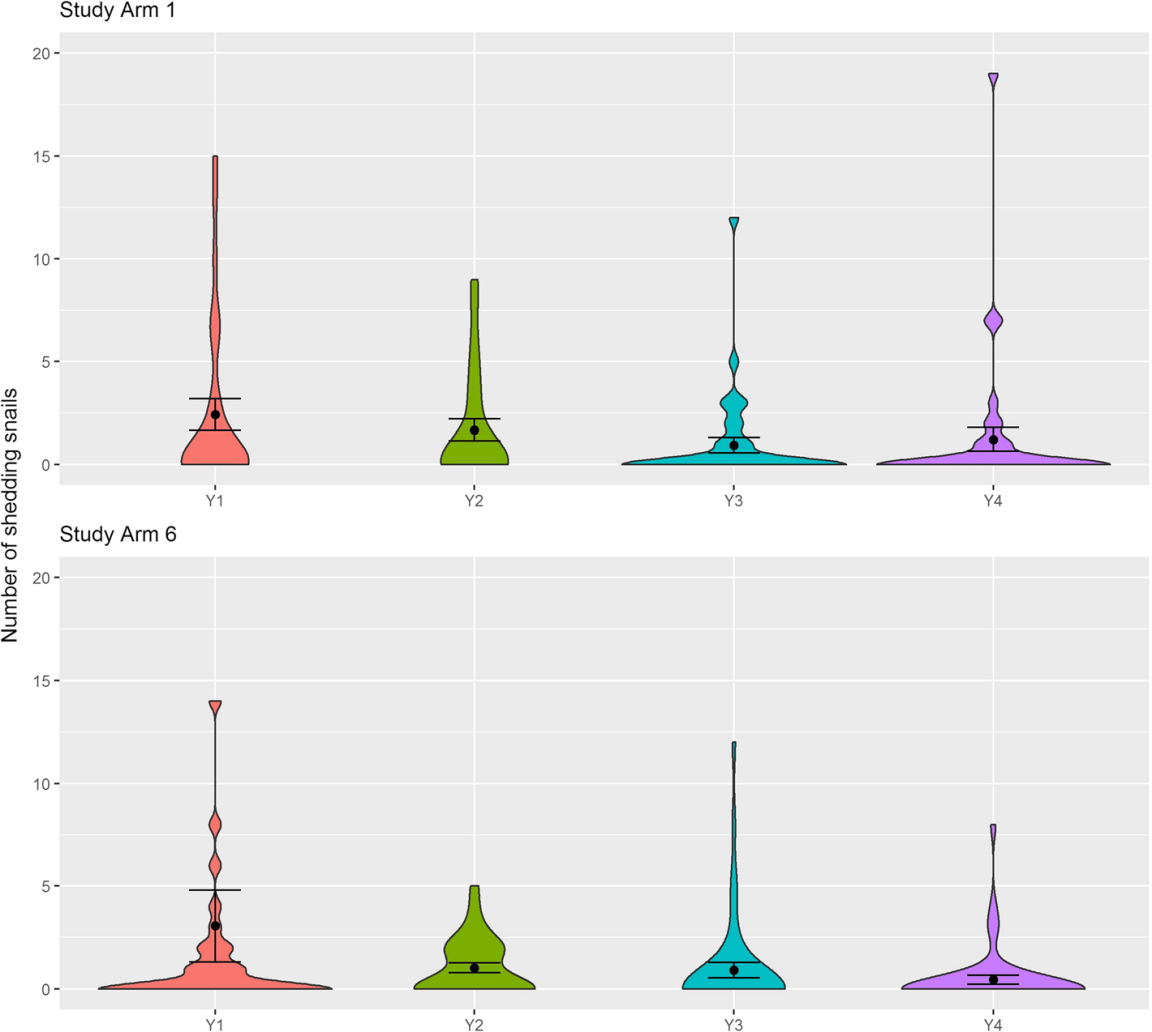
Violin plots including mean and standard error of mean of *S. mansoni*-shedding Biomphalaria* by study arm and year. Study Arm 1: Annual community-wide mass drug administration; Study Arm 6: Biennial school-based mass drug administration. *Outliers not shown: 58 shedding snails in Study Arm 6 2012

Due to the small sample size of the other treatment strategies, only annual community-wide treatment strategy (Study Arm 1, 6 villages, 10 sites) and biennial school-based treatment strategy (Study Arm 6, 7 villages, 12 sites) sites were analysed (Fig. 6). For Study arm 1, no significant difference was found in the average number of shedding *Biomphalaria* at baseline compared to post-treatment years 2, 3 and 4 (Kruskal-Wallis *χ* ^2^ = 3.963, *df* = 3, *P* = 0.265). Conversely, a significant difference was observed for the average number of shedding *Biomphalaria* in Study Arm 6 (Kruskal-Wallis *χ* ^2^ = 11.347, *df* = 3, *P* = 0.010). However Welch’s ANOVA could not confirm the findings of the Kruskal-Wallis test (*F*_(3, 75.715)_ = 1.704, *P* = 0.173).

#### Shedding *Biomphalaria* snails by year

**Fig. 7:**
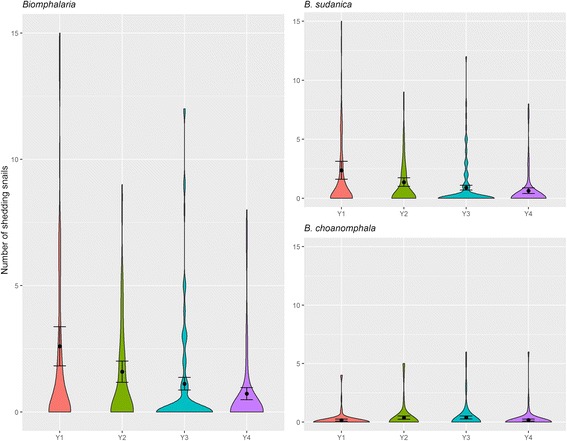
Violin plots including mean and standard error of mean of *S. mansoni*-shedding Biomphalaria* snails (all), B. sudanica* and *B. choanomphala* snail species by year (Y1: 2012; Y2: 2013; Y3: 2014; Y4: 2015). *Outliers not shown: 58 shedding snails in year 1 (2012) and 27 shedding snails in year 2 (2013)

Yearly median abundance of shedding *Biomphalaria* snails was significantly different (Kruskal-Wallis *χ* ^*2*^ = 21.282, *df* = 3, *P* = < 0.0001). A *post-hoc* test indicated that Y1 and Y4 were significantly different (Kruskal-Wallis *post-hoc* testing, *P* < 0.05) as shown in Fig. 7. However Welch’s ANOVA could not confirm the findings of the Kruskal-Wallis test (*F*_(3, 173.55)_ = 2.509, *P* = 0.060).

#### GLMMs of shedding *Biomphalaria* species

As no shedding snails were found at Katunguru throughout 4 years of survey, we assumed the sites were not transmission sites. We analysed the count data with and without the Katunguru sites and no significant difference was found between them; therefore, we chose to remove Katunguru from the binomial models.

The best zero-inflated negative binomial GLMM and binary binomial GLMM included *B. sudanica* abundance, *B. choanomphala* abundance, average monthly temperature, 4 weeks accumulative precipitation, presence/absence of human water activity at site, presence/absence of farming at site, presence/absence of other molluscs at site, and year of survey (Additional file 1: Table S4).

Abundance of shedding *Biomphalaria* was positively associated with abundance of *B. sudanica* [coefficient effect: 0.384, 95% CI (0.128, 0.641)] and with an interaction term between average temperature and 4 weeks accumulative precipitation [coefficient effect: 0.625, 95% CI (0.170, 1.080)] in the zero-inflated negative binomial GLMM. Fewer shedding *Biomphalaria* snails were found in 2015 compared to 2012 [Year 2015 coefficient effect: -1.086, 95% CI (-1.908, -0.263)]. The binary binomial model on the presence or absence of shedding *Biomphalaria* indicated *B. sudanica* abundance was positively associated with presence of shedding *Biomphalaria* [BS coefficient effect: 0.365, 95% CI (0.049, 0.681)]. Here also the 2015 group was negatively associated with the presence of shedding *Biomphalaria* snails compared to 2012 [Year 2015 coefficient effect: -1.396, 95% CI (-2.339, -0.453)].

## Discussion

In this study we conducted longitudinal field surveys at 26 sites in the Mwanza region of Lake Victoria basin, Tanzania, to identify *S. mansoni* transmission sites and to determine the parameters that influence the abundance of the snail intermediate host and of *S. mansoni* transmission.

Our results show that *Biomphalaria* species are present all year round most likely due to stable climate in the Mwanza region and the permanency of Lake Victoria. Figure 2 shows the typical bimodal rain pattern and stable temperatures of the Mwanza region. However, when we compared *Biomphalaria* abundance between seasons we did find seasonal variations with abundance of snails being greatest during the dry season (July, August and September). We analysed this further for the two species of *Biomphalaria* found in Mwanza.

### *Biomphalaria sudanica* and *B. choanomphala* abundance

*Biomphalaria sudanica* was present at all surveyed sites whereas *B. choanomphala* showed much more variability. The latter species is thought to be influenced by the lake substratum, preferring mud and sand and either low or no vegetation and little silt or decaying vegetation [15]. Whilst *B. sudanica* was ubiquitous across sites, *B. choanomphala* was found only in certain sites and particularly in Luhama.

The highest numbers of snails were collected during the dry season (June–September) whereas the lowest numbers of snails in the Masika Long rains (Fig. 3). There was a significant difference between the mean seasonal abundance for *B. sudanica* but not for *B. choanomphala*. This impact of seasons on one species but not on the other makes sense when considering the inherently changing habitat on or behind the lakeshore of *B. sudanica* compared to the constant lake habitat of *B. choanomphala*. We hypothesised that this could be explained by *B. sudanica*’s preference for lower pH water [21], the high rainfall during the rainy season probably increases the pH level, especially in the *B. sudanica*’s preferred habitat - marshes on or behind the lake shore. However, the GLMM model on *B. sudanica* abundance shows that water properties measured (pH, TDS, water temp) have no discernible effect on *B. sudanica* snail abundance around Lake Victoria. There is a small effect of changing temperature, mainly a negative association, on the number of *B. sudanica* (Additional file 1: Table S1). Normal water level is associated with a higher abundance of *B. sudanica* compared to flooded water levels at sites. This could be due to a decrease of *B. sudanica* numbers or a decrease of *B. sudanica* density, making flooded sites with more dispersed snails harder to sample from. Indeed this is in agreement with Magendantz [15] who observed high densities of *B. sudanica* during the dry season and few *B. sudanica* in surveys done during the Masika long rains. One aspect to consider regarding water levels and rainfall is that precipitation measurements are consistently taken at the Tanzania Meteorological Agency, Mwanza Station which is located near Mwanza city. However, rainfall does vary across the area of our study with rainfall patterns in Geita district sites and Magu district sites potentially being different. This variation is missed by applying the Mwanza station precipitation measurement. Observed water level at time of collection is perhaps more suitable to assess impact of rainfall at each individual site; however, this is a more subjective measurement prone to observational bias and errors.

*Biomphalaria choanomphala* collected by lake dredges were not significantly more abundant during the dry season although they were more abundant in May and June compared to other months and least abundant in the Mvuli Short Rain months (Figs. 3, 4). Standley et al. [21] did not find an association between *B. choanomphala* and pH (which in this study was not measured from dredged water). However, Magendantz [15] reported that *B. choanomphala* does not like habitats with high levels of silt and decaying vegetation. One could hypothesise that during rainy seasons there is high water flow from the banks into the lake, increasing silt, decaying vegetation and turbidity, hence why *B. choanomphala* may thrive better in the drier months when there is less rainy season turbidity. Our Zero-inflated model and the zero-truncated hurdle model do indicate that high amounts of rainfall before snail collection is associated with lower numbers of *B. choanomphala* (Additional file 1: Table S2 & S3). This may also be due to increased turbidity in the water. In addition, looking at geographical locations of the site there was a significant negative effect of longitude on *B. choanomphala* presence and abundance and Geita district sites (western sites on Fig. 1) had the highest abundance of these snails and Magu and Misungwi the least (eastern sites on Fig. 1). This may be due to gradual substratum changes on the lake bed, which warrants further research.

### Shedding *Biomphalaria*

Our results show that certain sites maintain consistent transmission, whereas others vary over time. One site did not show any evidence of *S. mansoni* transmission, namely Katunguru. Yet the prevalence of schistosomiasis in the local school is high (49% prevalence in 2012 according to SCORE-NIMR data). We can only conclude that transmission was occurring elsewhere either on the lake or another water body away from the lake. The sites with the highest number of shedding *Biomphalaria* are Chole, Kigongo and Luhama, all busy sites with lots of human population movement (Fig. 5). Chole and Luhama are busy fishing villages, Chole also being an important fish market. Kigongo is one of the main ferry sites that transport commuters to and from Sengerema. Luhama is interesting because unlike Chole and Kigongo where *B. sudanica* is the main shedding species, both *B. sudanica* and *B. choanomphala* are involved in schistosomiasis transmission at Luhama, with more *B. choanomphala* shedding *S. mansoni*.

Although the Kruskal-Wallis and Welch’s ANOVA tests showed that *Biomphalaria* abundance is highest during the dry season, there was no significant difference in the numbers of shedding *Biomphalaria* between seasons. GLMMs indicate that the number of shedding snails was positively associated with abundance of *B. sudanica* indicating that *B. sudanica* is the main *Biomphalaria* species involved in schistosomiasis transmission in the Mwanza region. However, this does not apply to all sites; Luhama clearly showed that *B. choanomphala* is strongly involved in schistosomiasis transmission at this site. Whereas *Biomphalaria* abundance for both species did not change significantly by year, number of shedding snails was significantly lower in 2015 (Year 4) relative to 2012 (Year 1), (Fig. 7; Additional file 1: Tables S4). This could be linked to ongoing schistosomiasis treatment efforts in the neighbouring human populations. If so, it is of interest that it has taken at least 3 years to observe such a decline in number of shedding snails. This warrants further research including neighbouring school infection intensity and prevalence and local treatment strategy to see if there is an association between schistosome infection prevalence in the human population and number of shedding snails observed, or even better, number of prepatent snails as an indirect measure of water contamination. When looking at the decline in number of shedding snails by schistosomiasis treatment intervention strategy (Arm 1- Annual community-wide and Arm6 - Biennial school-based), there was a significant decline in number of shedding snails in year 4 of study Arm 6 but not in Arm 1 (Fig. 6). However, this could be because there was a higher number of shedding snails at baseline for Arm 6 sites than for Arm 1.

### Study limitations

Due to the focal and changing nature of water contact and snail-schistosome transmission sites it is difficult to identify all transmission sites around an area. Water contact sites can change due to agricultural practices, presence of wild animals such as crocodiles and hippos native to Lake Victoria, and new building constructions etc. There is a potential disadvantage of dredging as a collecting method; we do not know the topography under the water thus it is often difficult to know where exactly to dredge. Therefore, it is also hard to determine how effective dredging is as a collecting method for *B. choanomphala*. Infected *Biomphalaria* may have been missed since shedding was only tested over a period of 24 h. Molecular techniques to identify infected snails will be more useful in assessing schistosomiasis contaminated sites and potential schistosomiasis transmission sites. The analysis with GLMM could also be improved by using Markov chain Monte Carlo (MCMC) model for more accurate confidence intervals. Additional sites away from the lake, temporary ponds, and irrigation canals should also be inspected where possible as they may play seasonal roles in schistosomiasis transmission, particularly for *S. haematobium* transmission [14, 23].

## Conclusions

In this study, we report on longitudinal surveys on *Biomphalaria* snail species in the Mwanza region of Lake Victoria, Tanzania. The study revealed that there is a seasonal difference in *B. sudanica* abundance with highest numbers occurring in the dry season; however, this was not linked to variations in water properties but was linked to water-level at each site with less *B. sudanica* snails found in flooded sites. Should snail control be considered it may be best to aim for months when water-levels around the lake shore are low such as in the dry season. *Biomphalaria choanomphala* varied greatly across sites but generally decreased with increasing longitude and were negatively associated with increasing accumulative precipitation. Snail control targeting these harder to reach deeper-dwelling *Biomphalaria* species could be much harder to implement; however, their involvement in schistosomiasis transmission appears to be far more focal and site specific than for *B. sudanica*. There is the possibility of integrating WASH measures in areas where snail control may not be feasible. What is clear is that the sites with the highest amount of shedding *Biomphalaria* have high levels of human movement either for fishing or for commuting, similar observations have been made by Magendantz [15] and for *S. japonicum* in the Yangtze River in China [31]. Prioritising snail control at sites such as these (fish markets, ferry and commuting sites and major fishing sites) could reduce transmission in these high-risk areas. Interestingly a significant decline in shedding *Biomphalaria* was observed in the 4th year, potentially associated with the 4th year of local treatment interventions in the neighbouring human populations. Low-risk areas could receive standard biennial preventative chemotherapy or MDA whereas in high-risk areas such as trade and fishing sites and commuting ferry terminals, an integrated, combined approach using human schistosomiasis control, snail control and water, sanitation and hygiene (WASH) measures could have a significant impact on schistosomiasis transmission in the Mwanza region.

## Additional file

Additional file 1: Table S1. Negative Binomial GLMMs for *B. sudanica* abundance showing coefficient effects estimates and confidence intervals (CI) for the following explanatory variables: monthly temperature, water levels at sites, water properties (water temperature & pH), and accumulative precipitation. **Table S2.** Zero-inflated negative binomial GLMM for *B. choanomphala* abundance showing coefficient effects estimates and confidence intervals (CI) for the following explanatory variables: monthly temperature, accumulative precipitation and districts. **Table S3.** Hurdle models: truncated quasi-Poisson binomial for *B. choanomphala* abundance and binomial for *B. choanomphala* presence/absence with coefficient effects estimates and confidence intervals (CI) for the following explanatory variables: monthly temperature, accumulative precipitation and longitude and latitude coordinates. **Table S4.** Zero-inflated negative binomial GLMM for shedding *Biomphalaria* abundance and binomial model for presence/absence of shedding *Biomphalaria* with coefficient effects estimates and confidence intervals (CI) for the following explanatory variables: *B. sudanica* abundance, *B. choanomphala* abundance, monthly temperature, accumulative precipitation, presence/absence of human water-based activities (bathing, washing, water collecting), presence/absence of farming activities, presence/absence of other molluscs (other snails and bivalves), year of collection and an interaction term between average temperature and accumulative precipitation. In all tables the symbol * indicates a statistically significant effect of the coefficient estimate (*P* < 0.05) (XLSX 22 kb)

## Abbreviations

AIC: Akaike’s information criterion
ANOVA: Analysis of variance
CI: Confidence interval
CWT: Community wide treatment
Df: Degrees of freedom
F: *F*-statistic
GLMM: Generalised linear mixed models
GVIF: Generalised variance inflation factor
MCMC: Markov chain Monte Carlo
MDA: Mass drug administration
NHM: Natural History Museum, London
NIMR: National Institute for Medical Research
SBT: School based treatment
SCAN: Schistosomiasis Collection at the Natural History Museum
SCORE: Schistosomiasis Consortium for Operational Research and Evaluation
TDS: Total dissolved solids
WASH: Water, sanitation and hygiene.
